# Alzheimer's disease: insights from *Drosophila melanogaster* models

**DOI:** 10.1016/j.tibs.2009.11.004

**Published:** 2010-04

**Authors:** Aileen Moloney, David B. Sattelle, David A. Lomas, Damian C. Crowther

**Affiliations:** 1 MRC Functional Genomics Unit, University of Oxford, Department of Physiology, Anatomy and Genetics, South Parks Road, Oxford, OX1 3QX; 2 University of Cambridge, Department of Medicine, Addenbrooke's Hospital, Box 157, Hills Road, Cambridge, CB2 2QQ; 3 Cambridge Institute for Medical Research, Hills Road, Cambridge, CB2 0XY; 4 University of Cambridge, Department of Genetics, Downing Street, Cambridge, CB2 3EH

## Abstract

The power of fruit fly genetics is being deployed against some of the most intractable and economically significant problems in modern medicine, the neurodegenerative diseases. Fly models of Alzheimer's disease can be exposed to the rich diversity of biological techniques that are available to the community and are providing new insights into disease mechanisms, and assisting in the identification of novel targets for therapy. Similar approaches might also help us to interpret the results of genome-wide association studies of human neurodegenerative diseases by allowing us to triage gene “hits” according to whether a candidate risk factor gene has a modifying effect on the disease phenotypes in fly model systems.

## Biological homologies allow us to successfully model aspects of Alzheimer's disease in the fly

**Figure 1:**
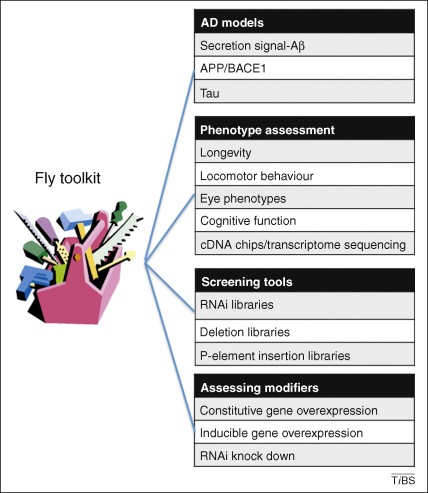
The fly provides a powerful toolkit for investigating the pathogenesis of AD. The range of AD model systems, and the fly phenotypes associated with them, allow investigators to screen of genetic and pharmacological modifiers of disease processes [32,36–40,43,44,47,58,77,88–95]. Several online resources are available for exploring the topic in greater detail: FlyChip: Functional Genomics for *Drosophila* (http://www.flychip.org.uk/) and NIG-Fly: National Institute of Genetics (http://www.shigen.nig.ac.jp/fly/nigfly/about/aboutRnai.jsp).

Many Alzheimer's disease (AD) researchers use animal models to gain insights into the pathogenic processes that occur in patients’ brains. In this review we will discuss why *Drosophila melanogaster* is a particularly powerful platform (Figure 1) and what we have learned from AD research in the fly. We will then discuss how the fly could become a tool in the interpretation of a new generation of human genetic studies.

Our faith in animal modelling is underpinned by a profound core of functional similarity that spans the phylogenetic classes. Indeed the degree of biological conservation from yeast to humans has surprised many investigators and is one of the most impressive findings to emerge from comparative genomics. Whereas in the pre-genome era we struggled to find similarities between organisms, now the biologist's burden is to define how such genetically similar organisms turn out so differently. Taking the number of genes as a crude measure of complexity, then the fly, *Drosophila* (13,767 genes) [1], is only slightly less complex than humans, who are now thought to have about 19,599 genes [2,3]. Bioinformatic approaches yield a critical core of biological similarity, and at the top of the list are the transcription factors and their target, non-coding DNA sequences [4]. These genes and DNA sequences are profoundly conserved across multicellular organisms; it has been said that at this level there has been precious little evolution since the appearance of bilateral symmetry [5]. Other gene families are also highly conserved across evolutionary time, including potential pharmaceutical targets such as the protein kinases and the homeobox domain proteins that play key roles in multicellular organisms [1]. This core of functionally essential genes is shared by both vertebrates and invertebrates, thus providing an explanation for why nearly 70% of human disease-causing genes have orthologues in the fly [1], and a similar proportion can be found in another invertebrate model system, the nematode worm, *Caenorhabditis elegans* [6]. It is likely that such conserved networks of interacting proteins and genes will respond in a similar way to a particular insult whether in a human or an invertebrate context (Box 1).

Although the specific details of protein–protein interactions can vary between insect and human, the degree of functional conservation can be surprising; of particular relevance for the AD field is the conservation of the proteolytic activity of γ-secretase between flies and humans. In humans it is proposed that AD is initiated by the dysfunctional activities of two proteinases (γ- and β-secretase) that generate a series of aggregation-prone peptides called Aβ from their substrate, amyloid precursor protein (APP). Excessive accumulation of Aβ peptides is thought to induce neuronal dysfunction and death. Conveniently, *Drosophila* γ-secretase can correctly process human APP, and a further similarity is that flies harbour an endogenous orthologue of APP called *Appl* (APP-like). Yet there is no natural generation of Aβ, as flies lack an equivalent of β-secretase and because APPL diverges in sequence from APP at the positions that constitute the Aβ peptides. Where generic physico-chemical interactions underpin a disease process, for example the proposed disruption of membrane integrity by protein aggregates, fly models might provide an excellent paradigm for research [7–9]. This exciting group of protein misfolding or conformational disorders includes the major human neurodegenerative disorders, including Alzheimer's, Parkinson's, Huntington's disease and forms of fronto-temporal dementia [10–12].

## Exposing molecular culprits

Although it is thought that AD pathology is initiated by the accumulation and aggregation of Aβ peptides [13], it seems unlikely that this accounts for all the features of the disease. Indeed, Aβ might not be required to maintain the disease process once it has begun. Aβ aggregation initiates a series of downstream events, including the phosphorylation and subsequent aggregation of tau within the cytoplasm. The combination of these Aβ- and tau-mediated [14] toxic events might result in the neuronal dysfunction and death that characterize AD [14–16]. However, the precise identity of the toxic species and their cellular targets remain elusive. Initial work on human brain tissue suggested that mature amyloid deposits of Aβ and tau were toxic; however, three key findings changed the opinion of many researchers in the field. First was the finding that amyloid plaque density did not correlate with clinical findings of AD, but instead, soluble Aβ levels [17] and tau pathology did [18]. Second was the observation that Aβ aggregates, intermediate in size between monomeric peptide and fibrils, were particularly toxic in cell culture and in *in vitro* models of synaptic function [19–24]. Third and surprisingly, it was possible to generate antisera that could bind the soluble aggregates of proteins, regardless of their amino acid sequence, and neutralize their cytotoxicity [25,26]. Notwithstanding the toxicity of Aβ fibrils under certain conditions [27], the weight of evidence points to small, soluble aggregates of proteins exerting a generic toxicity that is not entirely mediated by specific amino acid interactions, but rather seems to rely on shared biophysical interactions with cells [28].

**Figure 2:**
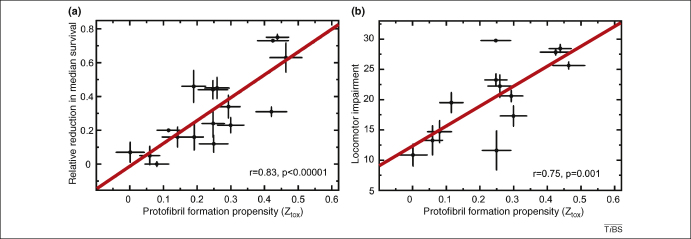
The propensity of Aβ variants to form protofibrils can be quantitatively correlated with fly model phenotypes. The expression of rationally designed variants of the Aβ peptide allows the investigation of the relationship between aggregation propensity and neuronal dysfunction and death in a fly model system. Here we see that the relative reduction in median survival **(a)**, and impairment in locomotor performance **(b)** are closely correlated with the computationally-predicted propensity of peptide variants to form protofibrils (Z_tox_) [7].

In this light, a body of research indicates that the fly can provide a faithful readout of the activity of the toxic protein aggregates. Guided by a computational approach to predicting aggregation propensity, Luheshi and colleagues undertook a systematic investigation of the sequence dependency of Aβ aggregation and toxicity [29–31]. To this end, they studied the *in vitro* behaviour of Aβ variants that had been designed rationally to have differing propensities to form either fibrils and/or protofibrils [7]. The neurotoxicity of these peptides was then tested using the fly model, and the predicted propensity to form amyloid fibrils correlated well with the *in vivo* toxicity, as measured by reduced longevity and locomotor performance. However a more satisfying correlation was shown between the predicted propensity of Aβ variants to form pre-fibrillar species and their *in vivo* toxicity (Figure 2). Indeed, the investigators were able to explain 80% of the variation in toxicity based only on knowledge of the primary structure of the polypeptide [7]. Interestingly, the correlation between phenotypic severity and the degree of peptide aggregation seems less strong when measuring age-related learning deficits in Aβ-expressing flies. Iijima and colleagues noted that similar learning phenotypes were observed in flies expressing Aβ_1-42_, which exhibited peptide deposits and neurodegeneration, and those expressing Aβ_1-40_, which had structurally intact brains [32]. This result points to a role for monomeric or very small soluble aggregates of Aβ in memory dysfunction.

The presence of such Aβ aggregates induces a series of responses that includes a transient influx of calcium and the activation of protein kinases. There is some suggestion that these responses might constitute an attempt by the neurones to re-enter the cell cycle [33]; however, the result appears to be the aberrant phosphorylation of a microtubule-binding protein called tau. The appearance of tau, phosphorylated at particular sites (as detected by specific monoclonal antibodies) has been observed to correlate with tau aggregation and its reduced affinity for microtubules [34], as well as with neurodegeneration. Recently the interaction of Aβ and tau in the fly brain was modelled, and it appears that Aβ enhances phosphorylation of tau by the wingless pathway component Shaggy (the fly orthologue of GSK3β [35]). With this report we can now aspire to a more complete reconstruction of the pathological pathways in the fly brain.

## What kinds of AD models have been generated in flies?

A particularly faithful invertebrate model of Aβ toxicity has been achieved by creating transgenic flies that carry gal4-driven constructs encoding human APP and human beta-site APP-cleaving enzyme 1 (BACE1). When expressed in the brain, human APP is cleaved by the transgenic human BACE1 and then by endogenous *Drosophila* γ-secretase, resulting in the generation of the Aβ peptide [36]. This relatively complex model is ideal for the assessment of modulators of BACE1 or APP metabolism, but, in some respects, is more cumbersome than models in which the Aβ sequence is fused downstream of a secretion signal peptide [32,37–39]. In these latter models, the expressed peptide has its signal peptide cleaved off as Aβ enters the secretory pathway and a proportion of the peptide is subsequently released from the cell. However, the degree of intracellular Aβ accumulation correlates with early phenotypes such as locomotor dysfunction [38] and severity, and immunogold electron microscopy reveals that the peptides localize to the endoplasmic reticulum (ER), Golgi and lysosomes, but not the nucleus or mitochondria [40]. This finding suggests that the potentially reversible early phenotypes in AD could be mediated by the intracellular accumulation and aggregation of Aβ.

Although Aβ-expressing flies can model one crucial aspect of AD pathogenesis, the role of tau is also of great importance. The tauopathies are a set of human neurodegenerative diseases related to AD, often presenting as fronto-temporal dementia, that are characterised by prominent intracellular accumulations of the microtubule-binding protein tau [41]. Familial tauopathies are caused either by deregulated mRNA splicing and the consequent accumulation of a particular tau isoform, or alternatively by an underlying genetic mutation [42]. Fly models allow us to investigate the mechanism of neurodegeneration in these tauopathies, but by extension, they also shed light on the role of tau in AD. The fly tauopathy models that have been generated thus far are tau-overexpression models. Although wild type human tau is neurotoxic when overexpressed in neuronal tissues, the rough eye and longevity phenotypes in *Drosophila* model systems are more severe when disease-related variants of tau are expressed [43] even when tau does not form neurofibrillary tangles [44]. Moreover, flies overexpressing wild-type human tau can be induced to form intracellular inclusions that resemble neurofibrillary tangles, when glycogen synthase kinase 3β (GSK3β) activity is increased [45]; this finding is concordant with the known pathways of tau toxicity that seems to require hyperphosphorylation of tau to speed aggregation.

## Which phenotypes and surrogate markers of pathology can be measured in fly models of AD?

**Figure 3:**
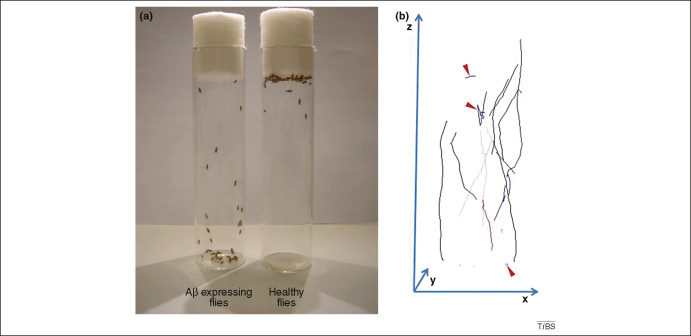
Quantifiable locomoter defects in flies expressing Aβ. Flies expressing Aβ peptides in their brains exhibit locomotor abnormalities **(a)**. Computer vision and 3D tracking of fly locomotor behaviour allows us to derive parameters that describe discriminatory features of fly locomotor behaviour. **(b)** The differently coloured traces represent the tracks followed by individual flies around the inner surface of a cylindrical culture vial. The trajectories followed by healthy flies are typically straight and oriented vertically, whereas Aβ expression results in age-dependent development of abnormal movement (similar to those traces marked with red arrowheads). Such analysis allows the early and rapid detection of the first signs of neuronal dysfunction and permits locomotor impairment to be quantified.

Longevity measurement provides a statistically robust test of the neurological integrity of a fly. Although the cause of death is not clear, it is probably related to a combination of behavioural deficits that impair feeding and hazard avoidance [46]. We have found that the fractional increase in median longevity as compared to control flies provides a parameter that has validity as a comparison between, as well as within, particular experiments [7]. However death is the last phenotype to be exhibited by an organism, and in a bid to accelerate data acquisition and to find experimental readouts that resonate with the clinical syndrome, there is a move to quantify and automate behavioural assays in flies (Figure 3). The most widely used behavioural assays that are employed are Pavlovian conditioning tests of memory and learning [32,40] and locomotor assays [32,38,46,47] (Please see supplementary movie).

In flies, locomotor assays such as the climbing test are popular because they need little equipment and they measure a clear phenotype. The locomotor assay is performed by placing the flies at the bottom of a tall cylinder and allowing them a specified time to climb before the number of flies at the top and bottom of the tube are counted and the ratio is expressed as a performance index [47,48]. These data give a single value for the locomotor performance, but do not represent the full complexity of the locomotor phenotypes. To measure this more fully there are a variety of video tracking technologies being developed that give either a two dimensional view of the fly movement [49], or track the fly in 3 dimensions [50,51]. By tracking flies we are able to calculate which parameters, such as maximal, mean and median velocity, best describe and distinguish control flies from those affected by model AD pathology. This approach will allow us to detect subtle changes in locomotor behaviour that characterize the early stages of neuronal dysfunction.

**Figure 4:**
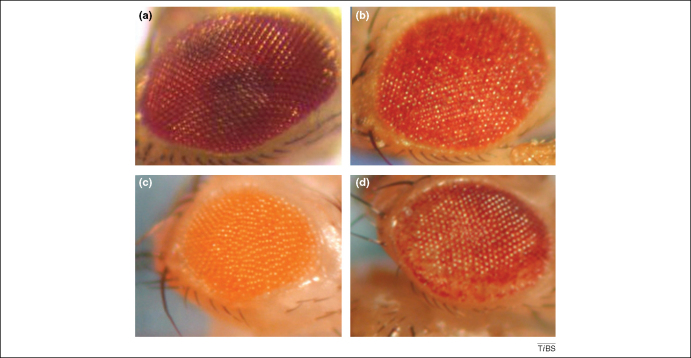
The rough eye phenotype provides a convenient surrogate marker for neurotoxicity. The normal compound eye **(a)** exhibits a regular array of corneal lenses (ommatidia) that are disrupted when toxic proteins are expressed during development. The assessment of eye roughness allows the investigator to look for modifiers of, for example, Aβ **(b)**, tau **(c)** and polyQ **(d)** toxicity [58]. Copyright is retained by the Genetics Society of America.

The rough eye phenotype is also easily recognized and has been particularly useful in the fly tauopathy models, where human tau expression in the retina yields adult flies with rough, shrunken eyes [43,52]. Likewise, expression of polypeptides containing expanded tracts of glutamine residues (polyQ), as a model of neurodegenerative diseases similar to Huntington's disease, results in a distinctive, rough, de-pigmented eye [53–55]; in both cases the clarity of the phenotype has facilitated genetic screening (Figure 4). The severity of the rough eye can be graded by a blinded observer and has the major advantage of being essentially fully apparent at the time of hatching (eclosion). Whereas the rough eye is not a direct measure of neuronal integrity, the pseudopupil assay provides an alternative way to assess the structural integrity of the underlying retina and follow progressive neurodegeneration [56,57]. The pseudopupil approach provides a quantitative measure of neurodegeneration, but is rather labour intensive; this property could limit throughput for whole genome screening.

## Large scale genetic screens in the study of Alzheimer's disease?

The use of genetic screens in the fly models of AD has been focussed on understanding the biological pathways by which dysregulated Aβ and tau production and aggregation might cause neuronal dysfunction and death. In the following sections we will review what these studies have taught us about AD pathogenesis.

### Screening for genetic modifiers of Aβ peptide toxicity

Genetic screening in the fly has been used to dissect the response of the fly brain to the insult posed by Aβ expression. Two such screens have been published, both in flies expressing Aβ fused downstream of a secretion signal peptide. In the first screen, Cao and colleagues looked for modifiers of the rough eye phenotype that accompany Aβ peptide expression [58]. Although the consequent phenotype is not strong (as compared to flies with excess tau or polyQ expression) it has the advantage of being quick to assess and has the dynamic range to allow the detection of both enhancers and strong suppressors. The investigators screened for dominant modifiers that resulted from, in large part, the over-expression of genes in a library of flies with 1963 unique insertions of mobile, transposon constructs (EP elements) that enhance the expression of neighbouring genes. In the second screen, Rival and colleagues expressed Aβ throughout the neurones, and used changes in longevity as the primary end point [47]. Although this assay is slow to perform, with the average fly living more than three weeks, the quantitative nature of the phenotype allows for the robust statistical interpretation of differences in median survival. Again the investigators used EP-like elements to generate the up-regulation of a random set of genes and were able to detect dominant modifiers. The biological implications of the two screens are somewhat different because, although in both cases the Aβ is expressed from early embryonic stages onwards, the rough eye is essentially a developmental phenotype, whereas differences in longevity are apparent later in adult life.

**Figure 5:**
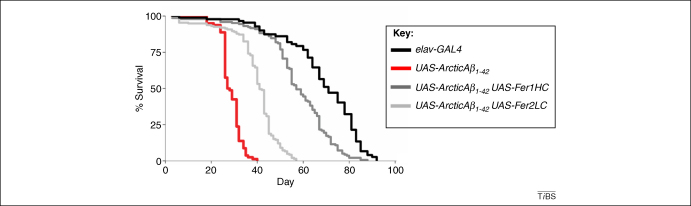
Genetic screens can be used to detect biological pathways that modulate disease-related phenotypes. Fly models systems can be used to discover novel genes that are involved in the expression of a disease phenotype. Following a genetic screen in flies expressing Aβ it was found that co-expression of the iron-binding proteins ferritin-1HC (dark grey) and ferritin-2LC (light grey) were the most powerful modifiers of Aβ toxicity. Here the data are shown for flies expressing the highly toxic Arctic variant of Aβ_1-42_ (E22G). The traces show that ferritin-1HC co-expression was able to significantly rescue the longevity phenotype and return the life span of Aβ-expressing flies (red line) back to almost control values (black line). Ferritin expression also rescued locomotor behaviour and oxidative stress measures back to almost wild-type levels [47].

Notably, both screens identified a role for the transition metals copper [58] and iron [47]. Rival and colleagues used an Affymetrix^®^ chip analysis to measure changes in gene expression in response to Aβ and reported that oxidative stress was a particularly significant contributor [47]. The most powerful modifying genes were those encoding iron-binding proteins; of particular note were the heavy and light chains of ferritin. Co-expression of ferritin heavy chain and Aβ suppressed the longevity and behavioural phenotypes (Figure 5) and reduced oxidative damage despite an increased accumulation of Aβ in the brain. A molecular dissection of the oxidative stress pathway highlighted the pathogenic role of the Fenton reaction in generating hydroxyl radicals. The ability of Aβ to induce a Toll/NFκB-dependent inflammatory response probably also increases the oxidative stress experienced by neurones [59].

### Screening for genetic modifiers of human tau toxicity

Screening for modifiers of the over-expression of human tau in fly models has been carried out by several groups. The most widely used phenotype in such screens is the rough eye that results from the retinal expression of wild type and variants of human tau. As the rough appearance is visible as soon as the adult fly hatches, this phenotype has been adopted widely as a surrogate marker of tau neurotoxicity.

There are, however, two main problems with the clear interpretation of the results from screens against tau toxicity. First, it is difficult to know exactly what toxic gain of function we are measuring. The two likely mechanisms are that either abnormal tau binding to microtubules causes their dysfunction or that excess unbound tau might remain in the cytoplasm and self-associate to form toxic aggregates. Alternatively, variant tau might trap proteins with essential cellular functions, and it is the loss of function of the binding partner that is the toxic event [60]. Secondly, the various tauopathy models are based on three or more disease-related variants of human tau (V337M, P301L and R406W). In the fly, both Shulman & Feany [43] and Blard and colleagues [57] have used the tau V337M variant because it gives a milder rough eye phenotype than either the moderate phenotype induced by wild type tau over-expression or the more severe phenotypes associated with R406W [61]. When these screens are compared, each shows that the phosphorylation economy of the cell is involved. This is particularly notable in the work by Shulman and Feany who find that three kinases and four phosphatases comprised the largest functional grouping in their set of 24 modifying EP-insertion fly lines. The precise message from this screen is complicated by the fact that two of the kinases enhance, whereas one kinase (par-1) suppresses, tau toxicity. Likewise, three of the phosphatases suppress, whereas one enhances, toxicity. The suppression of tau toxicity by par-1 is surprising given that the human orthologue of par-1, MARK (MAP/microtubule affinity-regulating kinase), binds neurofibrillary tangles [62] and is thought to promote toxicity by specific tau phosphorylation events [63]. One interpretation of this discrepancy is that at concentrations of tau found in the human brain, the par-1-mediated phosphorylation reduces the affinity of tau for microtubules, thereby resulting in toxicity [34]. By contrast, in the context of the over-expression of tau in animal models, tau might excessively bind microtubules, with phosphorylation relieving this effect. This argument cannot be applied to flies overexpressing R406W tau, because they show increased toxicity when par-1 is co-expressed; this result, however, might stem from the atypical response of this variant to phosphorylation. Specifically R406W tau is neurotoxic in murine model systems, despite being hypophosphorylated, in comparison to control mice expressing wild type human tau [64,65].

The screen performed by Blard and colleagues [52] identified a tyrosine phosphatase (Ptp4E) as a modifier of tau toxicity; however phosphorylation status was not a major functional group. Indeed, this disparity between screens is remarkable. Blard speculated that this might be due to differences in the screening protocol, because in their hands, over-expression of the candidate kinases par-1 and GSK-3β yielded a rough eye phenotype, and so would have been excluded from their analysis. The Blard screen instead emphasised the importance of cytoskeletal components. In particular, *cheerio*, the fly orthologue of the actin-binding protein filamin, was identified as an enhancer of tau pathology, in agreement with the findings of Shulman and Feany [43]. They also showed that tau over-expression causes morphological abnormalities in larval neuromuscular end plates which can be rescued or enhanced by the modifiers in the screen. This finding is of particular interest because there is mounting evidence that synaptic dysfunction is one of the earliest pathological events in AD [23,66].

In both of these screens the investigators also tested whether the modifiers of tau toxicity might have a broader activity against protein misfolding diseases. Therefore, they looked for modification of the eye phenotype caused by another disease-related cytoplasmic protein. In both cases the investigators crossed the tau-modifier lines to flies expressing a toxic poly-glutamine expansion in proteins that are linked to two forms of spinocerebellar ataxia (SCA). Shulman and Feany found that their tau modifiers, on the whole, did not have an effect on the eye phenotype in a model of SCA-1 [43]. By contrast, Blard and colleagues did observe some overlap between the modifiers of tau and polyQ phenotypes. However it appears that the shared modifiers act in distinct ways in the two model systems. For example, the three chaperones (DnaJ-1, Csp and Hsc70Cb) that enhanced V337M tau toxicity had a variety of effects on their SCA-3 flies such that DnaJ-1 was a suppressor, Csp was an enhancer, and Hsc70Cb had no effect [52].

## Concluding remarks and future perspectives

AD, like many of the common neurodegenerative diseases, shows a high degree of heritability; indeed 60-80% of the risk in so-called sporadic AD is genetic [67]. The existing human genetics studies, however, have explained only half of this risk. The main contributions are from the apolipoprotein E [68] and clusterin loci [69,70], and to a lesser extent from *PICALM* [69], *CR1* [70] and *SORL1* [71]. The heritability that remains unaccounted likely stems from a large number of genes, each of which provides a small contribution to disease risk. The current generation of genome-wide association studies are designed to detect these small contributions by exhaustively linking single nucleotide polymorphisms (SNPs) with risk for disease across hundreds of thousands of loci per individual [68,72].

With this vast amount of data for each subject, the statistical power of these studies is enormous, and we are able to identify large numbers of genes that are involved in the pathogenesis, along with some false positives. The task of prioritizing these long lists of possible human modifier genes is labour intensive and there is a need to focus detailed studies on those genes that have fundamental roles to play in the disease process. Herein could lie the next application of invertebrate model systems and of course this work will not be confined to *Drosophila. C. elegans* is also a model for AD and many other neurodegenerative diseases [73] with an equally useful genetic toolkit [74,75]. The use of such systems allows us to assess the pathological importance of large numbers of possible modifier genes (several hundreds), particularly where worm or fly orthologues exist. Genes that are found to have a functional importance in worms and flies, as well as showing linkage to disease in humans, will be of particular interest for future detailed studies. This approach has been facilitated by the whole genome-scale RNAi libraries now available for worms [76] and flies [77]. Any of the proposed human orthologues that show modifier activity in the worm can then be rapidly investigated in *Drosophila* models prior to studies in mouse. Detailed behavioural assays, increasingly assisted by automation and computational processing of video data [50,51], will allow us to find genes that have a phylogenetically conserved role in mediating tau and/or Aβ toxicity, and might therefore prove to be fundamentally important steps in the pathogenesis of AD. Moreover, these fundamentally important gene products will be the targets for a new generation of therapeutic compounds for the treatment, or even prevention, of AD.

## Box 1 Similarities between fly and human nervous systems

The biological similarities (otherwise known as orthologies) between humans and *Drosophila* have been exploited with great success in the field of neurodegenerative disease in general [78], and in AD research in particular. The principal reason behind this is that the fly has a brain, containing approximately 200,000 neurones, and like the vertebrate central nervous system, it is composed of a series of functionally specialized substructures. The primary sources of sensory input are visual and olfactory, and these are processed in the optic and antennal lobes, respectively [79]. The mushroom bodies deal with memory [80], and the central complex provides the motor output, once sensory integration is complete [81]. The functional units of the brain, the neurones, are also very similar to their human equivalents in terms of their shape, synaptic intercommunications and their biochemical signatures. These functional and structural similarities allow fly models of human disease to complement the rodent paradigms at the biophysical, molecular biological, neurobiological and behavioural levels. There are now fly models for Huntington's disease [55,57], a range of related polyQ expansion disorders [82], transthyretin-linked amyloidotic polyneuropathy [83,84], Parkinson's disease [85], motor neurone disease [86] and spinal muscular atrophy [87].
