# Supplementary figures and images for: Alzheimer's disease: insights from Drosophila melanogaster models
Source: Trends Biochem Sci. 2010 Apr;35(4):228–35. doi: 10.1016/j.tibs.2009.11.004 (PMC2856915; doi:10.1016/j.tibs.2009.11.004)

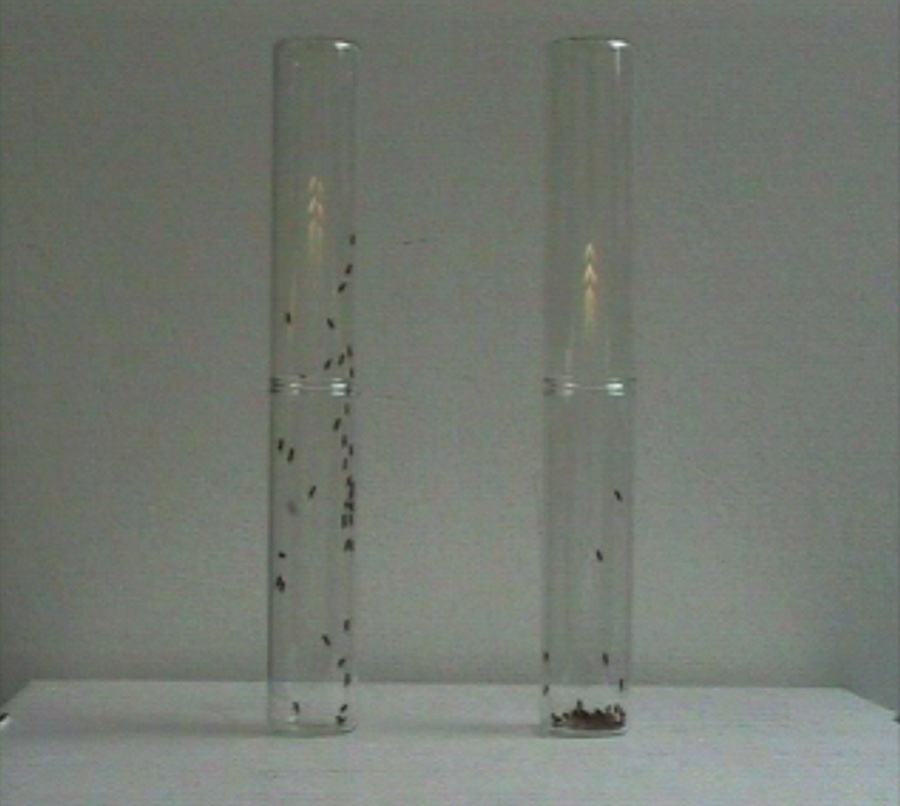

Supplement: Supplementary file 1 [file mmc1.jpg]
